# Normalization Methods for the Analysis of Unbalanced Transcriptome Data: A Review

**DOI:** 10.3389/fbioe.2019.00358

**Published:** 2019-11-26

**Authors:** Xueyan Liu, Nan Li, Sheng Liu, Jun Wang, Ning Zhang, Xubin Zheng, Kwong-Sak Leung, Lixin Cheng

**Affiliations:** ^1^Department of Critical Care Medicine, Shenzhen People's Hospital, The Second Clinical Medicine College of Jinan University, Shenzhen, China; ^2^Department of Stomatology Center, Shenzhen People's Hospital, Second Clinical Medicine College of Jinan University, Shenzhen, China; ^3^Department of Computer Science and Engineering, The Chinese University of Hong Kong, Hong Kong, Hong Kong

**Keywords:** normalization, microarray, RNA-seq, transcriptome, subset reference, regression

## Abstract

Dozens of normalization methods for correcting experimental variation and bias in high-throughput expression data have been developed during the last two decades. Up to 23 methods among them consider the skewness of expression data between sample states, which are even more than the conventional methods, such as loess and quantile. From the perspective of reference selection, we classified the normalization methods for skewed expression data into three categories, data-driven reference, foreign reference, and entire gene set. We separately introduced and summarized these normalization methods designed for gene expression data with global shift between compared conditions, including both microarray and RNA-seq, based on the reference selection strategies. To our best knowledge, this is the most comprehensive review of available preprocessing algorithms for the unbalanced transcriptome data. The anatomy and summarization of these methods shed light on the understanding and appropriate application of preprocessing methods.

## Introduction

The aim of normalization methods for large scale expression data, including microarray and RNA-seq, is to eliminate systematic experimental bias and technical variation while preserving biological variation. Dozens of normalization methods for correcting non-linear experimental differences between arrays have been developed during the last two decades (Dillies et al., 2013). Among them, quantile (Bolstad et al., 2003) and lowess (Berger et al., 2004) are well-adopted for analyzing microarray expression data. For the RNA-seq data, which generates short reads from fragmented RNA molecules and the reads number is proportional to the abundance of the transcripts (Ledford, 2008; Fu et al., 2009), the most frequently used normalization methods are “Reads Per Kilobase of transcripts per Million mapped reads” (RPKM) (Mortazavi et al., 2008) and Trimmed Mean of M-values (TMM) (Robinson and Oshlack, 2010). Some methods like quantile (Bolstad et al., 2003) and median normalization (Anders and Huber, 2010) are also employed for RNA-seq expression data, although these methods originate from the usage of microarray (Zhou et al., 2015a; Sun et al., 2019).

### Invalidated Conditions

Although a large number of normalization methods for high-throughput expression data have been proposed, most of which assume that (i) a majority of genes are equally expressed in each experimental unit and (ii) symmetrical distribution of genes between over-and under-expressed (Quackenbush, 2002; Robinson and Oshlack, 2010; Lovén et al., 2012). Hence, the application of these methods results in samples of similar or even identical distribution of gene expression intensities, which may not be biologically correct in many cases, e.g., arrays under comparison show unbalanced or global shifts in the transcripts population. Here we summarize the necessary conditions against the basic assumptions:

- When arrays under comparison are collected from different tissues or developmental stages. Different tissues contain different amounts of RNA. For instance, embryonic stem cells and fibroblasts show a significant difference in mRNA levels (around 5.5-fold; Aanes et al., 2014). Also, cancer cells generally contain more total RNA than normal cells and unbalanced gene regulation is common when investigated cancer samples (Lovén et al., 2012; Wang et al., 2012; Cheng et al., 2016a).
- A fraction of genes is enhanced or suppressed in the same direction as occurs in interspecific hybridization. When arrays are applied to strains that are inconsistent with the strain used to design the array, the probe intensities are all relatively lower than the standard strain at polymorphic sites.
- Small arrays tailored to specific applications or designed for a small-scale molecule, such as miRNA (Wu et al., 2013), of which the gene or feature number is too small to satisfy the statistical hypothesis (Handschuh et al., 2018; Wang et al., 2019). Usually, all of the features detected in small arrays are crucial to specific purposes and thereby they are likely to change in some specific patterns.

All of these three conditions, either under natural or experimental conditions, invalidate the critical assumption of equal expression levels between arrays under comparison. Instead, the total impact of biological variation and the percentage of genes containing gene effects are expected to be substantially large, which may to some extent mislead the downstream statistical inference and biological interpretation (Lovén et al., 2012; Cheng et al., 2016a). Notably, the realization of this issue has boosted the development of normalization methods for the shifted expression data. More than 20 methods, which are free of or based less on the basic assumptions, have been proposed during the last two decades (Table 1).

**Table 1:** A summary of 23 normalization methods developed for unbalanced transcriptome data.

**No**.	**Method**	**Full name or description**	**Platform (Original)**	**Reference selection**	**Normalization method**	**Software**
1	GRSN (Pelz et al., 2008)	Global rank-invariant set normalization	Oligo array	Subset	Lowess	R
2	Xcorr (Chua et al., 2006)	Cross-correlation normalization	cDNA array, Oligo array	Subset	Non-linear regression	Matlab
3	NVSA (Ni et al., 2008)	Non-parametric variable selection and approximation	Oligo array	Subset	Non-linear regression	Matlab
4	KDWL (Hsieh et al., 2011)	Kernel density weighted loess normalization	Oligo array	Subset	Loess	SAS
5	KDQ (Hsieh et al., 2011)	Kernel density quantile normalization	Oligo array	Subset	Quantile	SAS
6	IRON (Welsh et al., 2013)	Iterative rank-order normalization	Oligo array	Subset	Loess	C
7	LVS (Calza et al., 2008)	Least-variant set normalization	Oligo array	Subset	Non-linear regression	R
8	LVSmiR (Suo et al., 2010)	Modified least-variant set normalization	Oligo miRNA array	Subset	Non-linear regression	R
9	Invariants normalization (Pradervand et al., 2009)	Invariants normalization	Oligo miRNA array	Subset	Non-linear regression	R
10	HMM-normalization (Landfors et al., 2011)	HMM assisted normalization	Microarray, RNA-seq	Subset	Further subset normalization	R
11	BSN (Aanes et al., 2014)	Biological scaling normalization	RNA-seq	Subset		R
12	SVR (Fujita et al., 2006)	Support vector regression	cDNA, Oligo array	Subset	Non-linear regression	
13	ISN (Li and Hung Wong, 2001)	Invariant set normalization (in dChip)	Oligo array	Subset	–	R
14	Spike-in controls (Choe et al., 2005; Lovén et al., 2012)	Spike-in standards	Microarray, RNA-seq	Negative controls	–	–
15	wlowess (Oshlack et al., 2007)	Weighted lowess normalization	cDNA array, Oligo array	Negative controls	Loess	R
16	wcloess (Wu et al., 2013)	Weighted cyclic loess normalization	Oligo miRNA array	Negative controls	Loess	R
17	SQN (Wu and Aryee, 2010)	Subset quantile normalization	Oligo array	Negative controls	Quantile	R
18	loessM (Risso et al., 2009)	loessM	two-color miRNA array	Entire set (median)	Loess	R
19	GPA normalization (Xiong et al., 2008)	Generalized procrustes analysis	cDNA array	Entire set (median)	GPA	
20	Non-normalization (Klinglmueller et al., 2011; Wang et al., 2012)	Using data that are background adjusted but not normalized	Oligo miRNA array	Entire set	None	R
21	WPRMA (Kim et al., 2007)	RMA using within-pedigree pool	Oligo array	Entire set	Quantile	R
22	CrossNorm (Cheng et al., 2016a)	Cross normalization	Oligo array	Entire set	Quantile	R
23	ICN (Cheng et al., 2016b)	Informative cross normalization	Oligo array	Entire set	Quantile	R

### Normalization Methods for Unbalanced Data

For the normalizations in the case of being global shifted and unbalanced, they need a reference set of transcripts of genes that expected invariant or not vary intensely between samples. The underlying idea of selecting invariant features as a reference subset for normalization originates from using housekeeping genes for preprocessing qPCR data (Suo et al., 2010). Most conventional methods make use of the whole set of genes as the reference set, such as quantile and loess. With regard to the reference selecting strategy, the normalization methods for skewed expression data can be divided into three categories, i.e., data-driven reference, foreign reference, and the entire gene set.

- Data-driven procedures. A subset of genes that do not vary or vary least across samples is first identified as the data-driven housekeeping genes to train processing model. Genes whose array-to-array variability is below a given threshold are regarded as the reference set.
- Extra negative controls. External native controls have been designed in a number of experiments, such as spike-in probes. These native controls can be used as the foreign reference for signal correction.
- All genes in an array. Several algorithms consider the whole genome as a reference like quantile normalization, but they measure samples according to different references. Each compared sample group generates its own reference.

We briefly review the main features of each method in the listed categories above. Table 1 summarizes the characteristics of each method, including original platform, core algorithm, and available software. Hopefully, this is helpful for the readers who are interested in using tools for normalizing unbalanced expression datasets.

Generally, two types of techniques, microarray and RNA-seq, are used for quantifying the expression level of genes in high-throughput. Microarray normalization methods can be roughly categorized as single-color, two-color, and miRNA ones, although the two-color platforms are rarely used currently. Since the unbalanced expression pattern is frequently detected in certain conditions regardless of the selection of technique, we do not describe normalization methods from the aspect of techniques or platforms. However, the original platforms these methods designed for are listed, even practically all of which claimed its potential of trans-platform. Overall, we give a brief overview of 23 normalization procedures proposed for unbalanced expression data, including microarray, miRNA array, and RNA-seq, with a concentration on the reference selection in this survey. We wish to provide some basic recommendations for researchers to carry out the analytical steps. Further, a comprehensive evaluation of these methods is in process and some possible improvements on each step will be studied in order to present a more powerful normalization method.

## Brief Reviews of Available Methods

The three main categories of normalization methods, namely (i) data-driven procedures, (ii) external controls, and (iii) all-gene reference, are reviewed in the following sections Data-Driven Reference Normalization to All-Gene Reference Normalization, respectively.

### Data-Driven Reference Normalization

Methods in this category are generated based on the idea of rank-invariant transcripts presented by Li and Hung Wong (2001). They are designed to first identify an Invariant Transcript Set (ITS) expressed consistently across all arrays as a reference for further normalization in a dataset. Then non-linear regression methods, e.g., loess, lowess, or variance stabilizing normalization (VSN), are employed to fit a smooth curve for each array only using the data-driven reference. Finally, the fitted curve is used to map intensities of all the genes of each array, such as the horizontal line of the MA-plot. Unlike the conventional normalizations, this type of methods is expected to be applicable in most of situations as they require fewer or even no assumptions.

#### GRSN

Global rank-invariant set normalization (GRSN) is based on the general idea of selecting rank-invariant transcripts (Pelz et al., 2008). Assuming the expression rank on each array is not affected by the technique artifacts, GRSN first identifies an ITS that roughly show the same rank order of expression intensity on each array. All transcripts are ranked on each array according to the summarized expression intensity. Then, transcripts with high-rank variance are removed iteratively, removing one fourth each time and performing four times by default. The underlying assumption is that a high proportion of DEGs can lead to a global shift of transcript rank order, so the shift could be reduced because the most DEGs are discarded after a couple of iterations. The remaining transcripts are defined as subset reference and named “Global Rank-invariant Set” (GRiS). After that, the trimmed mean of all arrays for each transcript is determined as a common reference array. MA-plots are generated to compare each array to this common reference. For each array, a lowess curve is fitted by comparing the GRiS intensities to that of the common reference array, and then each array is centered to the A axis of the MA-plot, where M equals 0. It is claimed that using the trimmed mean values of the GRiS as the reference provides a robust average across all arrays, which can keep the linearity of the normalized data and avoid affections of outliers, such as DEGs.

#### Xcorr Normalization

Cross-correlation has been widely used for pattern recognition (Barucca et al., 2015; Stone and Veatch, 2015). Suppose we have two discrete time sequences, cross-correlation measures the similarity between one sequence and shifted copies of one another as a function of the lag. Cross-correlation (Xcorr) is adopted for normalization, which makes use of peak matching to minimize the effects of DEG points located in the tails of the distribution in MA-plot, with the assumption that the distribution peak of normalized expression value should be closer to zero (Chua et al., 2006).

Suppose the template be the distribution of *M*-values t(M) calculated from normalized expression value on an array, then we can match the s(M, A) of the expression value for all transcripts within a particular range of *A*-values (intensity window) with the template. The Cross-correlation of s(M, A) with t(M) can be maximized by tuning the matching parameter m. Specifically, the exact steps are described as “the optimal m is the one that can maximize the following formula,

$$\begin{array}{l} J\left(m\right)=\int_{M1}^{M2}s\left(M-m,A\right)t\left(M\right)dM \end{array}$$

which is assigned as the normalization factor k(A), where M1 is the lower bound and M2 is the upper bound of M component. In a MA-plot, all points are first segmented into n windows according to their *A*-values. Then s(M, A_i_) and K(A_i_) are calculated for each window, e.g., *i* in this case. The final normalization factor k(A) is calculated by fitting a spine function according to all k(A_i_).”

#### NVSA

Non-parametric Variable Selection and Approximation (NVSA) was developed for normalization of Affymetrix microarrays with a substantially large fraction of DEGs (Ni et al., 2008). NVSA operates a strategy of unique peak selection to identify and ITS prior to non-linear curve fitting. In a MA-plot, the method fits kernel density to the M component to points within an intensity window on the A component. The mode of one-dimensional kernel estimation is used to represent the bias of effect size on non-DEGs within each window, and the normalization curve is generated by connecting the modes across a series of windows of A with smoothing splines. NVSA is a complex algorithm with complicated program execution. It requires several individual steps and heuristic settings as well as several empirical parameters to select modes. Besides, its performance depends heavily on seed selection.

#### KDWL

Unlike NVSA measuring density solely on the M component, Kernel Density Weighted Loess normalization (KDWL) jointly fits kernel density in both the M and A components. loess regression is then applied to generate normalization curves locally for all data points in the MA-plot (Hsieh et al., 2011). When fitting the loess curve, points close to each other along the A component collectively determine the curve trend and different weights are assigned to the data points merely according to the estimated kernel density. The author applied the estimated kernel density to the power of 4 as the weight of the corresponding transcript, whereas transcripts that are far from the major group are down-weighted. This weighting strategy allows the normalization process to rely on the ITS heavily, although all of the transcripts are considered throughout the process.

KDWL only assuming that non-DEGs are distributed more closely than others. But the Golden Spike experiment violates this requirement as which includes two types of non-DEGs, unchanged transcripts, and empty transcripts. First, a common reference is generated using the average expression value for each transcript over all the arrays. Each array is normalized against the reference by fitting the weighted loess curve to the MA-plot using the A component as reference.

#### KDQ

Kernel density estimation (Fu et al., 2005) can also be exploited to select ITSs, including both non-DEGs and null genes. Accordingly, the conventional normalization method, such as quantile normalization, can be adapted for asymmetric data using kernel density estimation. Kernel Density Quantile (KDQ) normalization was proposed for this purpose and it typically consists of the three steps, ITS selection, quantile normalization with the ITS, and scoring the non-ITS transcripts (Hsieh et al., 2011).

To select the ITS, kernel density estimation is conducted to the MA-plot between the common reference array and each individual array. Each individual array or the average intensity of all arrays could be simply selected as the common reference array. As with KDWL, the A component represents the common average among all arrays while the M component was assigned as the variation between each individual array and the reference array. The density estimation score represents the relative importance of each data point in the MA-plot. The ITS was defined as the set of transcripts with high scores. After that, quantile was used to normalize the ITS for all arrays, and then the transcripts in the variant set are scored according to the ITS. Finally, “linear interpolation is applied within each array for the invariant transcripts whereas linear extrapolation is adopted for the other transcripts based on a small set of data at the boundary of the invariant set of transcripts.” This size of ITS is predefined but quite sensitive and data-dependent. It is recommended to be set lower than but close to the unknown percentage of non-DEGs.

#### Iron

Iterative Rank-Order Normalization (IRON) performs a pairwise normalization for each array against a common reference array iteratively (Welsh et al., 2013). For each pairwise normalization, a piecewise linear fit is implemented against an ITS. The fitted normalization curve is then used to non-linearly scale the array intensities. A common reference array is selected by first calculating root mean squared distance (RMSD) (Jewett et al., 2003) between a given array and all the other ones. The array with the smallest sum of RMSD is chosen as the common reference array and is used throughout normalization. After that, each array is processed independently. First, a set of probes is identified as ITS to train the normalization curve. Iterative rank-order pruning is then performed together with the common reference array to eliminate the most highly rank-divergent probes, until a convergence of 1% rank-invariance. The remaining points are regarded as the ITS and are simply scattered on the X and Y-axis representing the compared arrays, rather than the regular MA-plot. These data points are sorted by log(X^*^Y), where X and Y are the intensity on the reference array and the sample array to be normalized. A sliding window is then used to train weighted least-squared lines of log(X/Y) and log(X^*^Y). Finally, every transcript is normalized using the final correction factor relative to the nearest point on the fitting curve.

IRON iteratively identifies the training set for fitting regression curve and the common reference array is selected by implementing all possible comparison between arrays. It is emphasized that the fitting step outperforms the commonly used loess regression.

#### LVS

The least-variant set (LVS) normalization for mRNA arrays models a function considering both array effect and probe effect (Calza et al., 2008). The procedure contains two steps. Transcripts with the smallest array-to-array variation, called LVS transcripts, which is quite similar as ITS, are selected first as the reference set for further normalization. The step of selecting LVS transcripts allows the estimation of the component of variance due to array-to-array variability. The second step involves a non-linear fit of the LVS transcripts from individual arrays against those from a reference array. Once the LVS transcripts are identified, the normalization algorithm fits a smooth spline between the individual arrays and a reference array. Finally, the LVS-fitted smooth spline is used to scale intensities of all the transcripts in each array. The reference array is usually set as a pseudo-median array or any user-specified array.

#### LVSmiR

As with miRNA arrays, the transcript volume is much smaller than that of mRNA arrays. Therefore, the basic assumptions are not satisfied and traditional mRNA array normalization methods may not fit miRNA arrays well. However, some procedures originally applied on mRNA platforms with global shift could be extended to the miRNA framework, such as the LVS normalization (Calza et al., 2008). LVSmiR is an adapted version of the LVS normalization for miRNA arrays, which has a more complex model to identify the least variant set (LVS) of miRNA (Suo et al., 2010). The model is based on a linear model fitting of the probe level data considering the high effect of miRNA, but it requires several parameters to make ideal inferences from the data to choose the low-variance miRNAs (similar as ITS) as reference for normalization. LVSmiR normalization first fit a linear model for the raw data to estimate the component of variance due to between-array variability for each probe. Based on a quantile regression of the between-array variability vs. the residual standard deviation, the algorithm selects a set of low-variance miRNAs among arrays. Then these miRNAs are used to normalize each array to a reference array using either a variance stabilizing normalization (VSN) or a smooth spline. LVSmiR exploits all information at the probe level by simply computing the array-to-array variability accounting for the heterogeneity of probe-to-probe variances within a miRNA.

#### Invariants Normalization

Pradervand et al. developed an algorithm to normalize miRNA array named Invariants normalization, which first selects invariant miRNAs and then uses them to compute linear regression normalization coefficients using a mixture model of the mean and variance distributions (Pradervand et al., 2009). The invariant miRNAs (ITS of miRNAs) are defined as ones with medium-high mean intensity and low variance across arrays. (1) Considering background correction, the log intensity of each array was centered on the modal value of its data density distribution, because a large number of miRNAs are expressed at a very low level or even not expressed, and its modal value of the intensity distribution corresponds to that of all miRNAs on the array. (2) To identify ITS of miRNAs. It first removes the standard deviation (SD) vs. mean trend and then selects invariant probes from the mean and corrected SD. All the data points in the SD-mean plot are used to fit a loess curve that corresponds to the trend of SD as the function of the mean. (3) For each array, transcript intensity values were scaled using regression coefficients that were obtained from an M estimator with Huber influence function with default tuning constant. This algorithm avoids a large proportion of probes near or at the background signal level and just assumes a model-based low-SD/high-mean population. The loess curve before the removal of the SD vs. mean trend is indicative of the between-array variability.

#### HMM-Normalization

HMM-normalization is a type of invariant method using a Hidden Markov Model (HMM) (Ghavidel et al., 2015) to identify a set of non-DEGs that can be jointly operated with the standard normalization methods (Landfors et al., 2011). The workflow consists of four steps: (1) Normalize the raw data using one standard method, such as quantile. (2) Calculate the average intensities of the compared groups as well as the *M*-value for each transcript. Identify DEGs and detect whether its distribution is skewed using the Detection of Skewed Experiments (DSE) test. (3) An HMM with two states, variant and invariant, is applied to the *M*-values. Transcripts with the minimum absolute value of mean M are determined as ITS. (4) Normalize the data using a standard normalization only based on the ITS.

#### BSN

RPKM (Mortazavi et al., 2008) and TMM (Robinson and Oshlack, 2010) are the most commonly used normalization methods for RNA-seq. Similar as microarray studies, these algorithms are based on the basic assumptions. However, biological scaling normalization (BSN) tries to retain biological differences between arrays and assumes that RNAs detected at a particular stage would have a global upward shift (Aanes et al., 2014).

BSN takes advantage of polyA+ RNA amounts as scales to normalize the RNA-seq data. The transcript concentration and the average library size are two important parameters. A pseudo library size is represented by the product of the average library size and a stage specific scaling factor that can be obtained mathematically or experimentally. After that, the pseudo library sizes are assigned to each transcript based on the previously estimated expression value to obtain the normalized data. BSN is generated from the TMM normalization and they have similar main ideas, i.e., both of them have scales to represent the change in total expression. Specifically, the TMM scales use trimmed means of M-values on the read counts, whereas the BSN scales were based on measurements of polyA+ RNA content per embryo. The TMM scales are merged when estimating transcript concentrations, while BSN conducts this step later.

#### SVR

Support Vector Machine (SVM) can be adopted to both classification and regression problems (Zhou et al., 2019). Similar to loess normalization, Support Vector Regression (SVR) normalization take advantage of the regression algorithm of SVR to normalize microarray data (Fujita et al., 2006). One of the key ideas in SVR is that presenting the solution using only a small subset of training data points and hence it is extremely efficient. Existence of the global minimum and optimization of reliable generalization bound are guaranteed using the epsilon intensive-loss function. The SVR can fit reliable regression curves because it is not sensitive to DEGs and the selected training data points could be considered as an ITS. Generally, it is also a type of data-driven regression method.

#### ISN

The ISN procedure selects the reference genes in a pairwise fashion, which is implemented by identifying non-DEGs with a consistent rank between each array and a reference distribution, e.g., a pseudo-median array (Li and Hung Wong, 2001). It is expected that a probe of a non-DEGs in two arrays to have similar ranks in terms of intensity. ISN uses an iterative procedure to select non-DEGs that are the basis for fitting a normalization curve. Specifically, the running median curve is piece-wise linearly fitted in the scatterplot of probe intensities of two arrays, where Y-axis represents the baseline array and X-axis represents the array to be normalized. Then all the data points in the array on X-axis are adjusted to get the normalized value, while Y-axis is not changed as it is the baseline array. For each data point, its intensity on the Y-axis is assigned as the value of the fitted curve according to the specific intensity on the X-axis. Iteratively selecting rank-invariant gene set between any pair of arrays may reduce the phenotype effect between normal and cancer samples.

### Extra-Control Reference Normalization

Normalization methods developed for unbalanced array data usually first train regression curves using data-driven Invariant Transcript Set (ITS) that are expected to produce consistent measurements across arrays. Better still, ITS could be achieved by using the foreign controls, if the controls are designed to be embedded on the arrays. Several types of external controls have been developed for this purpose, including housekeeping genes, spike-in controls and microarray array pool (MSP) controls (Chua et al., 2006). The intensities of the negative controls are not affected by biological factors, but systematic factors affecting the entire array, such as labeling efficiency, scanner setting, batch effect, hybridization and washing conditions. Therefore, they are ideal reference for normalization and their intensities are expected to be constant across arrays even in the case of huge biological variation.

#### Spike-in Controls

External control technologies have been developed as a replacement for housekeeping genes. Spike-in experiments are expected to be the leading strategy to establish current normalization schemes, not only due to its powerful performance but also for its simplicity, involving only a few spike-in genes (Choe et al., 2005; Lovén et al., 2012).

In this approach, several mRNA transcripts with a series of intensities are spiked in arrays with equal amount. These spiked transcripts play a role as “anchors” that are ideal normalization features. For microarray, this technique first uses the MAS5 procedure to summarize raw probe intensities and obtain the expression values at the probe set level (Bolstad et al., 2003). Then, for each pair of arrays, these expression values are fitted using the loess regression only considering the spike-in probe sets. For RNA-seq, on the other hand, the RPKM was computed first for all the transcripts, including both real transcript and control transcript (spike-in RNA). Similar as microarray, a loess curve was then fitted on the RPKM values by using only the spike-in values for each pair of experiments. Finally, an expression matrix was generated with the entry indexes normalized RPKM values.

The spike-in technique is quite powerful to achieve relatively accurate measurements for experiments of both microarray and RNA-seq. A couple of modifications and then most of the existing normalization methods will work well for experiments with spike-in controls. However, the technique is not widely used merely due to extensive preparation works.

#### wlowess

wlowess normalization is developed for custom-made boutique arrays that may contain only a small number of printed probes of particular interest (Oshlack et al., 2007). wlowess employs whole microarray transcript pool (MSP) probes to normalize boutique arrays, which is expected to be robust against the bias of probe selection at a different range of intensities. Unlike spike-in control probes, MSP probes do not require extra added RNA on the arrays. The algorithm applies all the probes for lowess normalization but MSP probes are assigned higher weight in comparison with the real gene probes. This approach modifies the lowess fitting model on MA-plot by assigning a series of weights to every probe, including both real gene probes and control probes. Control probes are assigned higher weight in comparison with real gene probes. It is stressed that wlowess is able to smoothly utilize any composition of control probes and gene probes regardless of the intensity-dependent basis.

#### wcloess

Unlike mRNA arrays, the data of miRNA arrays normalized by assumption-free methods may improve identification of truly down-regulated miRNAs as well as reduce detection of false positive discoveries of up-regulated miRNAs. The key idea of weighted cyclic loess (wcloess) is to normalize arrays employ cyclic loess normalization relying on the external control probes (Wu et al., 2013).

Cyclic loess is a non-linear method applied to arrays in pairwise fashion and can transform the transcript intensities on the M component on the MA-plot. As with the wcloess algorithm, distinct weights are attributed to control probes and real miRNA probes in the loess curves. Specifically, it gives an extremely lower weight to the miRNA probes while gives much higher weight to control probes on each array. The external non-miRNA probes and miRNA probes are assigned with a weight of 100 and 0.001, respectively. The other probes are attributed a weight of 1, including GC control, spike-in, and hybridization control.

Cyclic loess cyclically executes loess to normalize any possible pairwise combination of arrays. The process will be rather time-consuming as it may repeat for thousands of iterations when more than 50 arrays are investigated.

#### SQN

Subset quantile normalization is developed for array dataset with negative controls and is a modified version of quantile normalization. Subset quantile normalization (SQN) is the relative term of complete quantile normalization (CQN) (Bolstad et al., 2003); SQN makes the intensity distributions of the control probes equal whereas CQN makes the intensity distributions of the whole array identical (Wu and Aryee, 2010). As with SQN, the quantiles of the negative control probes on each array are used as “anchors” that should be constant among each array. Then probe intensities on each array are normalized according to their relationship to the quantiles of control probes on the same array. One limitation of SQN is it requires a series of embedded control probes. It is not powerful for arrays only have a few control probes because of the stability of intensity quantile.

### All-Gene Reference Normalization

#### Loessm (miRNA)

loessM simply scales expression data on the global median expression on the basis of loess, in contrast to the typical scaling for whole-genome arrays on zero, which loosens the basic assumption (Risso et al., 2009). The miRNA expression data were characterized by a large number of DEGs and often skew in one single direction.

Generally, loess related algorithms adopt a non-linear regression technique based on robust local regression on the array-array scale or the M-A scale. loess normalization first fit a smooth curve according to all data points in the MA-plot and then adjust the curve to the A component, so that all the data points are scaled toward zero on the M component, i.e., M equals 0. However, the scaling procedure performs poorly when most of the features are under-expressed or over-expressed. Scaling expression values toward the overall median expression values, rather than zero, is proposed in this paper. The “M” in loessM represents the median of M on the microarray experiment.

#### GPA Normalization (cDNA)

Generalized Procrustes Analysis (GPA) is a method of statistical analysis and is widely applied to normalize data shapes (Xiong et al., 2008). GPA consists of three transformations, i.e., translation, rotation, and scaling. The optimal transformation of the GPA procedure is the one that has a minimum sum of the squared deviation among corresponding data points in the MA-plot.

Specifically, GPA is used to minimize the deviation of transcript intensities among arrays. Firstly, a reference array is set as the median intensity of each transcript over all arrays. Then, in the MA-plot, data points in each array are translated in order to make their centroid point the same as that of the reference array. Then the data points of each array are rotated and scaled to obtain a minimum residual discrepancy with the reference array.

The transformations (translation, rotation, and scaling) are based on global optimization rather than local optimization. In terms of choosing the reference array, GPA normalization employs median values across all arrays as the common reference array, which is further verified to be a right choice and perform better than the individual array. Another advantage of using GPA for normalization over other methods is that it assumes nothing about data distributions, which makes it applicable for all types of data.

#### Non-normalized Data

This method stressed that normalization is not necessary for microarray studies for which traditional normalization methods may produce more false positive discoveries (Klinglmueller et al., 2011). Non-normalized data were compared to normalized data, in the context of a microarray titration experiment, which is designed for producing reliable biological data with a proportion of DEGs larger than what can be simulated using spike-in experiments. They provide an alternative way to study the robustness of conventional normalization methods against violations of the basic assumptions.

The titration experiment highlights some of the pitfalls of microarray data analysis and some evaluating measurements for normalization methods are provided. Non-normalized data provided higher accuracy and agreement in the titration experiment in comparison with the normalized data. Normalization procedures pose a tradeoff between accuracy and agreement as well as repeatability and power, when the processed array data contain a large partition of DEGs.

Wang et al. also suggested applying the raw data to identify DEGs as a complement to improve the power of DEG detection (Wang et al., 2012). As with non-normalized data, however, it is difficult to tell the difference between biological variation and the technical variation caused by batch effect, limited sampling, probe hybridizing conditions, or scanning power. So most of the researchers still claim normalization is a necessary step before downstream array analysis.

#### WPRMA (Within Pool RMA)

WPRMA assumes only family members within a pedigree share the same distribution and utilizes RMA to arrays within pedigrees separately (Kim et al., 2007). Performing RMA normalization to each pedigree pool separately allows for the same number of distributions as pedigrees, instead of only one reference distribution.

RMA eliminates technical variation using quantile normalization, which assumes that the transcript intensities on each array are from the same distributions and the intensity values are then normalized according to a reference distribution. However, the selection of optimal reference is quite artificial for family data in genomics studies.

The familial similarity within pedigree is fundamental in linkage analysis. Also, pedigree data are chartered by more homogeneity within pedigrees than between pedigrees for the studied traits. Therefore, traditional normalization methods may improperly adjust the trait values by imposing identical distributions across pedigrees, which ignores the pedigree property in linkage analysis. The procedure of normalizing arrays in each pedigree separately maintains the individual familial distributions.

#### CrossNorm

From the perspective of gene expression direction, CrossNorm (Cheng et al., 2016a) illustrates that the standard normalization methods usually reverse the expression direction of thousands of genes in cancers, whereas CrossNorm makes full use of the raw signal and detects the regulation direction more precisely. CrossNorm first divided the expression matrix into two submatrix, cancer matrix and normal matrix, with rows represent genes while columns represent samples. Then, the two matrixes were combined by samples to generate a cross-matrix. After that, a traditional normalization method, such as Quantile, was applied to process the cross-matrix. Finally, the normalized matrix was reverted to the original format.

CrossNorm guarantees the columns of the combined expression matrix have the same intensity distribution, which is exactly the basic assumption of the traditional methods. The shortcoming of this method is that it is very fit for the datasets with case-control paired samples, but time-consuming for the datasets without the matching information.

#### ICN

Informative CrossNorm (ICN) (Cheng et al., 2016b) performs the CrossNorm on the expression matrixes concentrating on the informative transcripts or genes, as a fraction of transcripts are not informative, some of which express insufficient or even not express in specific tissues. ICN selects informative transcripts using I/NI-calls (Talloen et al., 2007), which is able to efficiently eliminate false positives, such as noise and biologically irrelevant transcripts. Basically, ICN combines I/NI-calls and CrossNorm to cross normalize the informative transcripts. It can improve the statistical power for the identification of differentially expressed genes, since some transcripts or genes filtered out by I/NI-calls would be determined as significantly differentially expressed otherwise (Calza et al., 2007).

## Discussion

### General Steps for Normalizing Strewed Gene Expression Data

**Figure 1:**
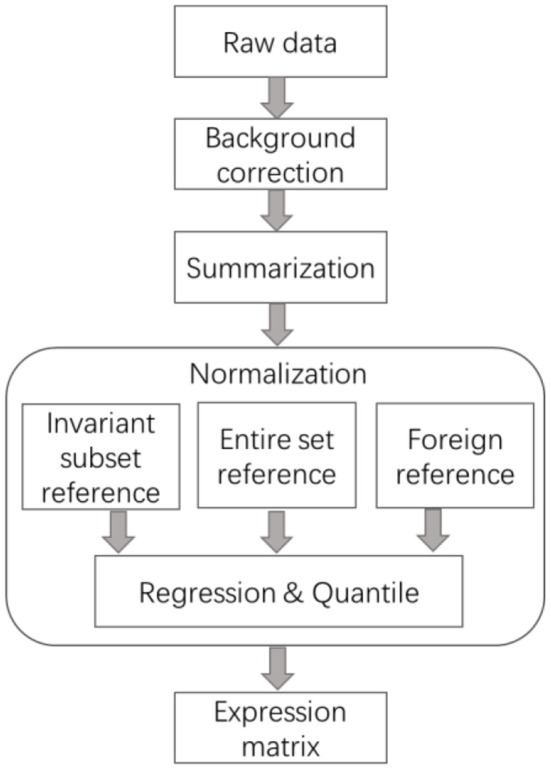
General steps of the preprocessing methods for skewed transcriptome data. The core step of normalization is the selection of reference, which was summarized into three categories, data-driven invariant subset, foreign subset, and the entire set. Sometimes summarization is after normalization.

The normalization steps, including identifying invariant transcript, selecting common reference, and regression, are of vital importance for all the preprocessing procedures of expression data. Figure 1 illustrates the general steps of processing the strewed gene expression data. It is noted that the global normalization methods developed for balanced expression data also follow these steps, like lowess and quantile, make use of the whole array as Invariant Transcript Set (ITS), instead of preselecting a subset, and then the intensities on each array are transformed using non-linear regression or scaling algorithms considering all the features.

### Optimal Hybridization of Existing Normalization Steps

For the step of identifying ITS or data-driven sub reference, 13 methods have been proposed, including GRSN, Xcorr, NVSA, KDWL, KDQ, IRON, LVS, LVSmiR, Invariants-normalization, HMM-normalization, BSN, SVR, and ISN. All of them based on distinct statistic models or kernel algorithms, such as GRiS in GRSN, LVS in LVS normalization, HMM model in HMM-normalization, etc. Four other methods based on external negative controls are quite similar to the methods in the invariant-set family, with the foreign controls as the predefined invariant-set instead of driving from data. lowess, SQN, wlowess, and wcloess are in this category. The other six methods, namely loessM, GPA, WPRMA, CrossNorm, ICN, and non-normalization, make use of all the features of an experiment and focus on the property of global shift rather than the ITS.

As with common reference selection, several approaches are applied among these normalization methods, such as the root mean squared distance (RMSD) between all array pairs, average expression value for each transcript over all the arrays, or the trimmed mean for each transcript among all arrays. When the ITS and common reference array are set well, non-linear regression approaches are implemented to adjust all the data points, such as loess, lowess, weighted lowess, piecewise spline, and LVS.

Therefore, a series of models and approaches have been conducted for normalizing a given expression data. A tuned combination of them is expected to show a higher performance, e.g., GRiS + RMSD + LVS, which is the combination of the three steps, identifying ITS, selecting common reference, and regression analysis. Consequently, an optimal hybridization of normalization steps is expected to maximize the power of these available methods.

### Potential Improvement of the Normalization Steps

Although more than 20 normalization methods have been developed for the skewed expression data, most of them have their own assumptions and suffer from similar problems. No real assumption-free methods exist but methods based on reasonable and adaptive assumptions are always highly required. Therefore, we stress that an in-depth understanding of data property is critical before conducing normalization. In addition, the interactions between different approaches among these steps should be noted. In other words, the result of one step may affect the other steps heavily, either the parallel or subsequent ones. Besides, it still calls for novel methods for RNA-seq data as for which limited solutions are proposed. Hopefully some existing methods originally developed for arrays can be employed after some sophisticated adaptions.

### Impact on lncRNA Transcriptome and Coexpression Analysis

The normalization algorithms we summarized are equally important for mRNAs and lncRNAs, because it is common to perform secondary analyses of lncRNAs by leveraging previously published gene expression data including both microarray and RNA-seq (Zhou et al., 2015a,b, 2017, 2018, 2019; Cheng and Leung, 2018a,b). In the present study, we studied the skewness between samples in different states, such as disease or normal, with a special emphasize. As for as we know, no normalization algorithms have been specifically developed for lncRNA expression data for this purpose. Recently, Assefa et al. summarized and compared a total of 25 methods for detecting differential expression lncRNAs concentrating on the low expression abundance (Assefa et al., 2018). They concluded that no methods compared can outperform other tools. For the differential analysis of lncRNAs, all tools exhibit substandard performance. They also concluded that large sample size is necessary for accurate differential expression inference of lncRNAs.

Normalization methods impact little to the coexpression analysis, mainly because the gene expression samples in different state or subgroups used to be stratified first and then perform the coexpression analysis. In this case, the skewness or difference between distinct biological states is not taken into account. Like the protein interaction network (Cheng et al., 2017a,b, 2018, 2019), module identification is also a common way for studying gene coexpression network. Similarly, normalization methods have little effect on the identification of gene coexpression modules. Overall, the impact of normalization methods on coexpression analysis is not as much as differential analysis.

## Conclusion

Normalization methods developed for the unbalanced expression data were summarized into three classes based on the expression reference, data-driven reference, extra negative controls, and all genes. To our best knowledge, this is the most comprehensive review of available preprocessing algorithms for the unbalanced transcriptome data. The anatomy and summarization of these algorithms shed light on the understanding and appropriate application of preprocessing methods.

## Author Contributions

LC and XL wrote the paper. NL, SL, JW, NZ, and XZ provided the literature and algorithms. LC and K-SL contributed to the overall paper design.

### Conflict of Interest

The authors declare that the research was conducted in the absence of any commercial or financial relationships that could be construed as a potential conflict of interest.
